# Load Position Estimation Method for Wearable Devices Based on Difference in Pulse Wave Arrival Time †

**DOI:** 10.3390/s22031090

**Published:** 2022-01-31

**Authors:** Kazuki Yoshida, Kazuya Murao

**Affiliations:** 1 Graduate School of Information Science and Engineering, Ritsumeikan University, 1-1-1 Nojihigashi, Kusatsu 525-8577, Shiga, Japan; murao@cs.ritsumei.ac.jp; 2 Strategic Creation Research Promotion Project (PRESTO), Japan Science and Technology Agency (JST), 4-1-8 Honmachi, Kawaguchi 332-0012, Saitama, Japan

**Keywords:** wearable device, wearable sensor, load position estimation, electrocardiogram (ECG), heartbeat, pulse wave, pulse wave velocity (PWV)

## Abstract

With the increasing use of wearable devices equipped with various sensors, information on human activities, biometrics, and surrounding environments can be obtained via sensor data at any time and place. When such devices are attached to arbitrary body parts and multiple devices are used to capture body-wide movements, it is important to estimate where the devices are attached. In this study, we propose a method that estimates the load positions of wearable devices without requiring the user to perform specific actions. The proposed method estimates the time difference between a heartbeat obtained by an ECG sensor and a pulse wave obtained by a pulse sensor, and it classifies the pulse sensor position from the estimated time difference. Data were collected at 12 body parts from four male subjects and one female subject, and the proposed method was evaluated in both user-dependent and user-independent environments. The average F-value was 1.0 when the number of target body parts was from two to five.

## 1. Introduction

Wearable devices equipped with various sensors are becoming increasingly popular. The sensors embedded in such devices can obtain biometric data and information about human activities and the surrounding environments at any time and place. These sensors can capture acceleration, angular velocity, light, pulse wave, position, radio wave strength, electromyograph (EMG) [1], electrocardiogram (ECG) [2], and galvanic skin response (GSR) [3] data, and other types of data obtained from manually configured sensors [4]. Such data can be used for a variety of services, such as health management systems that automatically extract life patterns and issue warnings about lack of exercise and overwork [3]. Another application is services that provide support during assembly and maintenance tasks [5] by predicting the next task from the current operation and presenting manuals and required tools. Support systems for diabetic patients record the times of insulin injections and provide blood sugar level measurements outside the hospital. Sports support systems acquire data on the number of times an athlete performs various maneuvers and activities (CATAPULT, https://www.catapultsports.com/ accessed on 29 January 2022). Entertainment systems capture data on changes in audience behavior [6].

The wearable devices currently on the market have various shapes and appearances, such as watches, bracelets, rings, glasses, belts, shoes, and clothes. Such devices are limited to a specific body part. However, devices that can be attached to any body part, such as an instant tattoo [7], a flexible sheet [8], and a general-purpose sensor [4], are also becoming available. When such devices are attached to an arbitrary body part, the location should be identified because the application process may need to change. In one such application, human activity recognition (HAR) systems change their machine learning algorithm according to the position of a device, because the recognition accuracy drops significantly when the training data domain differs from the test data domain [9]. For example, recognition of hand gestures by using a model that learned leg motions does not work well. The output method, such as a display, LED, sound, or vibration, can also change according to a wearable device’s position.

In recent years, mobile on-body robots have also been proposed. For example, Rovables move on clothes by using magnetic wheels [10], while the SkinBot moves on skin by using two suction cups [11]. In the latter case, body biopotentials (e.g., electrodermal activity and ECG signals) can be measured using electrodes inserted in the suction cups. Such wearable robots require self-position estimation, but SkinBot cannot localize itself. In contrast, Rovables perform localization through dead reckoning, a position estimation algorithm that determines the current position on the basis of the previous position, but this requires knowing the initial position.

For systems that use multiple wearable devices to capture body-wide movements, it is important to estimate the attachment positions. Assuming multiple identical devices, i.e., the same products for HAR, the devices can be connected to a PC via Bluetooth or a USB cable. Then, a COM port can be assigned to each device, e.g., “COM3”. The control system on the PC can identify devices from their assigned COM ports, but it cannot identify where they are attached. As users usually move body parts individually, the system can see a device’s waveform and then confirm the COM port associated with the body part. However, even if devices specify where they should be attached, COM ports often change when devices are connected.

Generally, studies on device load positions can be categorized as studies on displacement detection and compensation for a single body part and studies on load position identification for wearable sensors anywhere on the body. Studies in the first category enable a single recognition model to recognize human activities with compensated sensor values [12]. In these conventional studies, a small displacement (e.g., a few centimeters) can be compensated for; however, when the displacement is large or when a sensor is attached to a different limb (i.e., over a joint), the displacement cannot be compensated for, which results in low performance. As for the second category, studies on load position identification classify sensor load positions into predefined body parts [13,14]. However, these approaches require a certain amount of data as input to a classification model, and users must perform specific activities, such as walking for a few minutes.

In this paper, we propose a method to estimate the load positions of wearable devices without requiring the user to perform specific actions. The proposed method calculates the time difference between a heartbeat obtained via an electrocardiograph and a pulse wave obtained via a pulse sensor, and it then classifies the sensor position on the basis of the time difference. We assume that the pulse sensor is embedded in a wearable device. When the heart pumps blood to the body, the sinus node, a cluster of cells near the heart’s right atrium, controls constriction of the heart by electrical stimulation. An electrocardiograph measures the electric potential of this stimulation. Currently, small, coin-sized electrocardiographs and a clothing-type electrocardiograph are commercially available. The heart rate and various physical and mental characteristics can be acquired by analyzing ECGs [15,16], and, in the future, it is expected that users will be able to access personal ECG data on demand. In the human circulatory system, blood pumped from the left ventricle through the aorta (main artery) to the whole body passes through the arteries and reaches the capillaries. A pulse wave sensor can measure changes in blood flow. Here, the pulse wave arrival time depends on the distance from the heart, and as a result, the time difference between a heartbeat and a pulse wave measured by a sensor depends on the sensor’s location on the body. Accordingly, the proposed method detects the occurrence time of a heartbeat and the arrival time of a pulse wave, calculates the time difference, and used it to estimate the load position of the wearable device.

Note that part of this work was published at the 23rd International Symposium on Wearable Computers [17]. However, the data collected for the evaluation experiment reported in the conference paper was limited, with only one minute of data for each body part in an experimental session. Moreover, only a simple evaluation was conducted, with a session-dependent environment. Hence, for this paper, the evaluation experiment was repeated with data collection for 3 min at each body part in each session, and with two evaluation environments, which were user-dependent (i.e., session-independent) and user-independent. These environments were designed to reflect the actual use of the proposed method. As a result, this paper has been substantially revised from the conference paper [17].

The primary contributions of this study are as follows.

- The proposed load position estimation method does not require users to perform any specific activities: it can identify sensor load positions when a user stands still for 10 s;
- We assume that the sensor device whose load position is to be identified is equipped with a pulse sensor, which consists of an infrared LED and a photoreflector. Typically, such sensors cost less than half a US dollar and weigh less than a gram. Wearable electrocardiographs are also attached to the user, and their use here is relatively practical;
- In an experiment with five subjects, we collected ECGs and pulse waves at 12 body parts in four sessions lasting three minutes each. We then used the collected data to evaluate the proposed method in both user-dependent and user-independent environments.

The remainder of this paper is organized as follows. Section 2 introduces related work. The proposed method is explained in Section 3. The experimental evaluation is described in Section 4, and the limitations of this study are described in Section 5.

## 2. Related Work

This section introduces related work on sensor positions in HAR, position estimation for wearable devices, position estimation for smartphones, and ECG and pulse wave sensing.

### 2.1. Sensor Positions in HAR

Many studies have been conducted on recognizing human behavior by attaching multiple accelerometers to various locations throughout the body. This section introduces these studies and investigates various sensor locations.

Murao et al. [18] proposed an evaluation function that scores sensor placement in terms of both recognition accuracy and sensor wearability. This study involved 20 wearable sensors and 30 types of exercise including aerobic exercise, weight training, and yoga. In experiments conducted to evaluate sensor placement, the proposed evaluation function yielded an accuracy of 86% without inducing stress on the user.

Louis et al. [19] investigated differences in accuracy in HAR by having subjects attach three-axis acceleration sensors to seven locations: the ear, chest, arm, wrist, waist, knee, and ankle. Data were collected from 11 subjects who performed 15 different actions. From the collected data, the accuracy of action recognition was highest when the acceleration sensor was attached to the arm or ear. Gjoreski et al. [20] also had subjects wear three-axis accelerometers, at four locations (chest, waist, thigh, and ankle), and they investigated the optimal sensor locations for nine types of action recognition, including sitting and standing. Acceleration data were collected from 11 subjects, and classification was performed using features such as the mean and standard deviation. For the case of only one sensor, the results showed an average accuracy of 75% when the sensor was attached to the chest and 77% when it was attached to the waist; in contrast, the accuracy was insufficient when the sensor was attached only to the thigh or ankle. Furthermore, the accuracy increased to about 90% when the single sensor was combined with ankle data and 93% when it was combined with thigh data.

In another study, Cleland et al. [21] investigated the optimal sensor locations for behavior recognition by having subjects wear three-axis accelerometers at six locations: the chest, wrist, waist, buttocks, thigh, and ankle. Data were collected from eight subjects for seven different behaviors, such as walking and running, and the behaviors were classified using four different machine learning algorithms: a J48 decision tree, a naive Bayes, a neural network, and a support vector machine (SVM). The classification results showed an accuracy of 97.81% when the sensor was attached only to the buttocks. Finally, Pannurat et al. [22] investigated the optimal sensor placement for recognizing seven types of actions, such as sitting and lying down, by using data collected from 12 subjects aged 23 to 45. The results showed an average accuracy of 96% when the accelerometers were placed on the chest, anterior waist, lateral waist, and thigh.

### 2.2. Position Estimation for Wearable Devices

Several studies have reported that system performance drops if a classification model designed for a specific body part is used with a different body part. For instance, Apiwat et al. [23] asked 10 subjects to attach accelerometers to three positions (shirt pocket, pants pocket, and waist) and perform six different activities. Then, they evaluated activity recognition by using sensor data collected from different positions in the training and test data. When the waist data were used for both training and testing, the accuracy was 91.71%. However, when the waist data were used for training and the shirt pocket data were used for testing, the accuracy was only 51.58%. The results thus demonstrated that an appropriate classification model is required for motion recognition.

Vahdatpour et al. [24] used acceleration data to estimate the load positions of wearable sensors. In an experiment with 25 subjects, 10 accelerometers were attached to the head, both upper arms, both forearms, the waist, both thighs, and both shins, and acceleration data were collected during daily activities such as walking. The load positions were estimated from the collected data by using an SVM, an average accuracy of 84% was achieved. Timo et al. [25] collected acceleration data on human activities, such as walking and running, from 15 subjects who wore accelerometers at various positions, such as the head, chest, upper left arm, left wrist, waist, front shirt pocket, and left ankle. By using a random forest, the load positions were estimated from the collected data with an average accuracy of 89%. Kunze et al. [26] collected acceleration data from six subjects who walked with sensors attached to the right wrist, the side of the right eye, the left pants pocket, and the left shirt pocket. A C4.5 decision tree was used to estimate the load positions, and an average accuracy of 94% was achieved. Similarly, Takata et al. [13] had 10 subjects wear a device equipped with accelerometers and angular rate sensors at a total of 10 locations: the head, chest, left wrist, right wrist, waist, front pants pocket, back pants pocket, left ankle, and right ankle. They collected sensor data from the 10 subjects during daily activities such as walking. By estimating the load position with a random forest, they obtained a maximum accuracy of 98.2% and an average accuracy of 90.0%.

In a study on device displacement compensation, data were collected from one subject who had accelerometers attached to six points on the lower right arm [12]. The sensor load positions were identified from the data distribution with an average accuracy of 84%. Moreover, the results showed that a small displacement (e.g., a few centimeters) could be compensated for; however, when the displacement was large or the sensor was attached to a different limb (i.e., over the a joint), the effect could not be compensated for, and the performance was low.

### 2.3. Position Estimation for Smartphones

Several studies have estimated where mobile devices, such as smartphones, are being carried. For instance, Coskun et al. [27] used acceleration data to estimate the carrying location of a mobile device. Fifteen subjects carried a handheld terminal in three places: a hand, backpack, and clothes pocket. Acceleration data were collected during daily activities, such as walking and running, and a random forest was used to estimate the carrying location. Using only acceleration data, the average accuracy was 77.34%. When both acceleration and angular velocity data were used, the accuracy improved to 85% on average. Martin et al. [28] estimated the storage location of a mobile device by using data from five types of sensors: an acceleration sensor, gravity sensor, linear acceleration sensor, magnetometer sensor, and gyroscope sensor. By using a decision tree to estimate the storage location, an accuracy of up to 92.94% was obtained. Fujinami et al. [14] used acceleration data to estimate five storage positions during walking: the front pants pocket, back pants pocket, jacket pocket, breast pocket, and a neck strap. Acceleration data were collected for each position from 10 subjects, and an average accuracy of 72.3% was obtained.

Park et al. [29] also used acceleration data to estimate the location of a mobile device. Nine subjects were asked to hold or store their mobile devices at four locations (the hand, ear, backpack, and clothing pocket) and acceleration data were collected while the subjects walked at three different speeds. From collected data, they could estimate the mobile device’s location with an average accuracy of 94%. Maarten [30] estimated the locations of smartphones worn by pedestrians and cyclists. Forty-nine subjects carried their smartphones in four locations: the jacket breast pocket, front pants pocket, back pants pocket (or bicycle back pocket for cyclists), and backpack. Acceleration and angular velocity data were collected, the device location was estimated with an average accuracy of 79.5% for pedestrians and 90.5% for cyclists.

In another approach, Itzik [31] proposed a method that used deep learning to estimate the position of a smartphone carried by a walking user. Acceleration and angular velocity data were collected from 107 subjects who kept a smartphone in a pocket, near an ear (while talking), at waist level (while texting), and swinging in a hand (while walking). For training with a single user, the combined use of acceleration and angular velocity data yielded an accuracy over 95%, while the acceleration data alone yielded an accuracy over 91%. In comparison, for learning with multiple users, the combined use of acceleration and angular velocity data yielded an accuracy over 94.8%, and the acceleration data alone yielded an accuracy over 91.6%.

Most conventional studies have estimated the load positions of sensors and devices by using accelerometer and gyroscope data; accordingly, they required users to perform specific actions so that each sensor could generate values that were unique to the various positions. In contrast, pulse wave time differences always occur and vary with the distance that blood flows, which is unique for different positions. To the best of our knowledge, no study to date has used ECG and pulse wave data to estimate sensor load positions.

### 2.4. ECG and Pulse Wave Sensing

Many studies have used ECG and pulse wave data in various ways, including estimation of the peak time of an ECG and the arrival times of pulse waves. Assuming a situation in which an electrocardiograph cannot be attached but pulse wave sensors can be, Robert et al. [32] estimated the peak time of an ECG by calculating the time difference between pulse wave peaks. The peak time was estimated with an average error of 14.3 ms for standing and 9.43 ms for sitting. Rajala et al. [33] evaluated whether the time difference between ECG peaks, referred to as the R-peaks, and pulse wave peaks could be calculated using pulse waves measured from the wrist and fingers. Pulse wave data were measured from 30 subjects at rest. For calculation of the pulse wave arrival time at the wrist, it was found that using the first derivative peak method or detecting pulse wave arrival at the foot was suitable. In addition, the shape and amplitude of pulse waves were found to be different between the wrist and fingers.

There are also methods for estimating blood pressure values from ECG and pulse wave data. Yoon et al. [34] tested whether blood pressure could be continuously estimated without a cuff by using pulse wave analysis based on a multi-parameter model. The experiment used data collected from 23 subjects. With an average regression model, the resulting standard deviation of error was 8.7 ± 3.2 mm Hg for estimation of systolic blood pressure and 4.4 ± 1.6 mm Hg for diastolic blood pressure. The results thus showed a small standard deviation of error by using pulse wave analysis rather than pulse wave arrival times.

Liu et al. [35] also proposed a cuffless method for estimating blood pressure by using pulse wave data measured by a pressure sensor. Their method extracted 21 types of features and created a model by using a linear regression method. In evaluation of the model on 65 middle-aged and elderly subjects, they could reduce the error by 1.33 ± 0.37 mm Hg for systolic blood pressure and by 1.14 ± 0.20 mm Hg for diastolic blood pressure as compared with the conventional method. Simjanoska et al. [36] proposed a method for estimating blood pressure values by using only ECG data. They developed a machine learning algorithm that combined a stacking-based classification module and a regression module into a prediction model for blood pressure values. They collected ECG data from 51 subjects and could estimate the systolic and diastolic blood pressure with average absolute errors of 8.64 and 18.20 mm Hg, respectively. Furthermore, calibration based on a probability distribution reduced the respective errors to 7.72 and 9.45 mm Hg. Sun et al. [37] proposed a method for estimating systolic blood pressure by using ECG and pulse wave data. They collected ECG and pulse wave data from 19 subjects who exercised for 40 min, and the standard deviation of error for the estimated blood pressure values was 13.52 mm Hg.

These studies estimated biological information, such as blood pressure, vascular age, and ECG peaks from data obtained by sensors at known positions. To the best of our knowledge, however, a study on estimating the load positions of devices from time differences in pulse waves has not yet been conducted.

## 3. Proposed Method

This section describes the process of the proposed method.

### 3.1. Overview of Proposed Method

**Figure 1:**
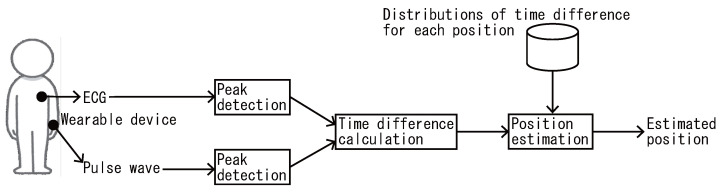
System flow of the proposed method.

As shown in Figure 1, the proposed method comprises four processes: ECG and pulse wave measurement, ECG and pulse wave peak detection, peak time difference calculation, and load position estimation.

Here, we assume that the user attaches a small electrocardiograph sensor to the chest, which provides constant measurements. In addition, a wearable device attached to an arbitrary body part contains a photoplethysmography (PPG) sensor and measures pulse waves at load positions. Note that the proposed method works in real time. From the start time to the current time, the device positions are identified from the ECG and pulse wave data. For example, given data acquired over 10 s, all peaks (e.g., 15 peaks) in the 10 s of ECG and pulse wave data are detected. Then, 15 time difference samples are calculated, and the device position is identified on the basis of these 15 samples. Given data from a longer interval, the identification accuracy should thus increase, because many time difference samples can be used and the time differences become stable.

The following subsections explain each of the four processes in detail. For simplicity, we assume a single wearable device; however, the positions of multiple wearable devices can be identified by individually applying the proposed method to each device.

### 3.2. ECG and Pulse Wave Measurement

First, the ECG data from the chest ECG sensor and the pulse wave data from the pulse wave sensor located at an arbitrary body part are collected. The raw sensor readings often contain noise due to the analog–digital converter and show jagged peaks. Accordingly, we apply a moving average filter over a 21-sample (≈26 ms) window, defined as $\sum_{i=t-10}^{t+10}f\left[i\right]$, to facilitate easier peak detection. Here, f[t] denotes the raw sensor readings at index *t*. After applying the moving average filter, the ECG (i.e., cardiac potential) and the pulse wave sensor output (i.e., the pulse wave) at *t* are denoted as $x_{h}\left[t\right]$ and $x_{p}\left[t\right]$, respectively.

### 3.3. ECG and Pulse Wave Peak Detection

**Figure 2:**
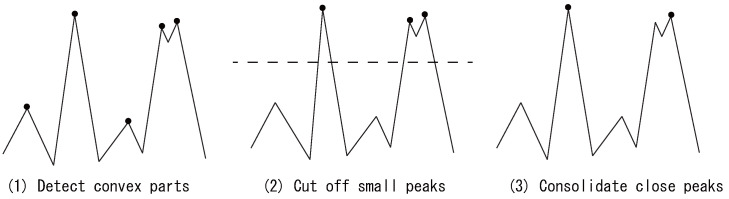
Example of peak detection: each • denotes a peak found during the process).

Next, the proposed method detects peaks in the ECG and the pulse wave, as illustrated in Figure 2. Given an ECG signal $x_{h}\left[t\right]\;\left(t=0,\cdots ,T-1\right)$, peaks are detected as follows.

(1) Find all convex parts that satisfy $\left(x_{h}\left[t+1\right]-x_{h}\left[t\right]\right)\times \left(x_{h}\left[t\right]-x_{h}\left[t-1\right]\right)\leq 0$. If the same values continue after a peak, i.e., if the top of a peak is flat, the midpoint of the flat interval is detected as a peak;
(2) Delete peaks below a threshold value defined as $\theta_{h}=\max\left(x_{h}\right)-\alpha \cdot \left(\max\left(x_{h}\right)-\frac{1}{T}\sum_{t=0}^{T-1}x_{h}\left[t\right]\right)$, where α is a coefficient that is empirically determined from the collected data; here, we set it to 0.5;
(3) Consolidate peaks that are close in time; this may be completed in order from the highest peak, for example. Any other peaks in the 0.2 s periods to the left and right, are deleted. This is completed because the peak interval even at 200 bpm (the maximum human heart rate) is 0.3 s; therefore, two true peaks will never appear within the same 0.3 s window.

Through this process, ECG peak (i.e., R-peaks) are found, and pulse wave peaks are detected by the same process. It is possible, however, that a pulse wave peak is not readily detected because of the contact state between the sensor and the skin. In that case, when no convexity is detected by the above algorithm, we define the peak as the maximum value $max(x_{p})$ of the pulse wave within the interval between the current ECG peak and next ECG peak. To finish this process, the indices of all detected ECG and pulse wave peaks are stored in $h_{peak}$ and $p_{peak}$, respectively.

### 3.4. Peak Time Difference Calculation

Here, the proposed method calculates the time differences between the detected ECG and pulse wave peaks. Blood delivered from the heart is detected as a pulse wave at each body part with a delay of tens of milliseconds. The proposed method finds the pulse wave peak corresponding to an ECG peak, because the ECG signal is more stable than the pulse wave. Fake peaks occasionally appear because of the contact state of the pulse sensor and the skin.

Beginning from index t=0, the proposed method finds a peak and calculates the time difference from the corresponding pulse wave peak. All time differences are obtained through this process until reaching the maximum index t=T−1. Here, we assume that there is an *n*-th ECG peak at $h_{p}eak\left[n\right]$, and we denote the time for this peak as ${tmp}_{h}\left[n\right]=timestamp\left(h_{p}eak\left[n\right]\right)$. We then find a pulse wave peak between ${tmp}_{h}\left[n\right]+0.25$ [s] and ${tmp}_{h}\left[n\right]+0.5$ [s]. The interval is set in this manner because we determined through preliminary experiments that the difference at any part of the body ranges from 0.25 to 0.5 s. Accordingly, by applying this interval, fake pulse wave peaks can be eliminated.

Next, let ${tmp}_{p}\left[n\right]=timestamp\left(p_{p}eak\left[n\right]\right)$ denote the time of the *n*-th pulse wave peak found by the above procedure. The time difference d[n] is then obtained as

(1) $$d\left[n\right]={tmp}_{p}\left[n\right]-{tmp}_{h}\left[n\right]$$

If an interval contains multiple pulse wave peaks, the highest peak is used. If there is no pulse wave peak, the time difference is not calculated for the ECG peak in question.

**Figure 3:**
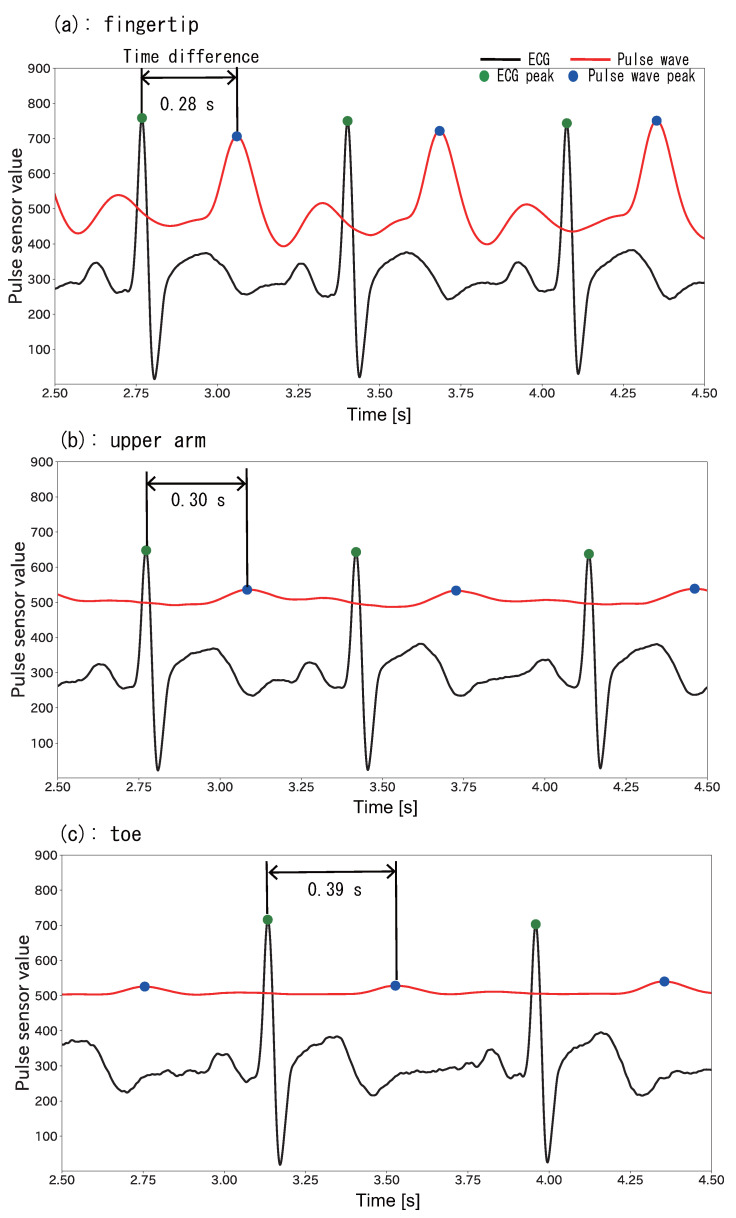
ECG and pulse wave peaks and time differences for (**a**) a fingertip, (**b**) an upper arm, and (**c**) a toe. The black line represents the ECG, and the red line represents the pulse wave. The green and blue circles indicate the ECG and pulse wave peaks, respectively.

Figure 3 shows ECG and pulse wave waveforms obtained at (a) a fingertip, (b) an upper arm, and (c) a toe. As seen in the figure, the time difference between peaks is different for each body part. For example, the time differences at (a) the fingertip and (b) the upper arm are 0.28 s and 0.30 s, respectively. In contrast, the time difference for (c) the toe, which is farther from the heart, is 0.39 s and thus longer. The proposed method uses this property to estimate the load position by obtaining, multiple time differences. For example, given 30 s of ECG and pulse wave data with an 80-bpm heart rate, there are 40 peaks in the data, and, 40 time difference samples are ideally collected.

### 3.5. Load Position Estimation

Finally, to estimate the load position, the proposed method calculates the distance between the set of time differences collected at each position in advance, which constitutes the training data, and the set of time differences obtained at unknown positions, which are the test data. For the distance calculation, we use the Kullback–Leibler (KL) divergence [38], which is a measure of how a probability distribution differs from a reference distribution. A KL divergence of 0 indicates that the two distributions are identical.

**Figure 4:**
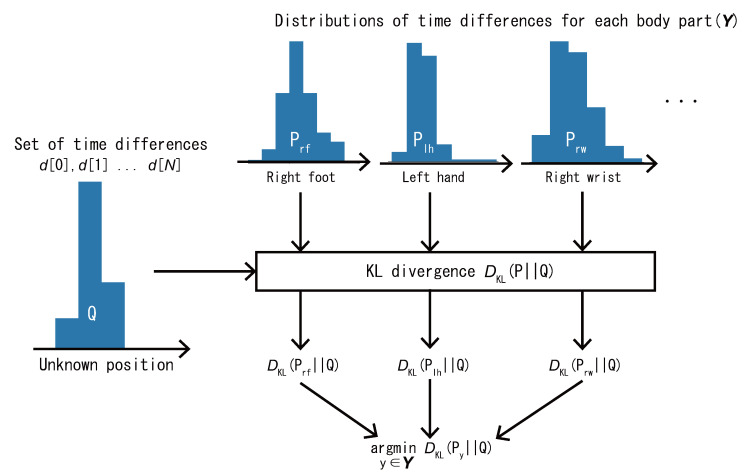
Position estimation based on the KL divergence between the training and test data distributions.

Figure 4 shows the process flow for load position estimation by using KL divergence. Note that the calculation requires a distribution for each dataset. Here, histograms ranging from 0.25 to 0.5 s with a bin of 0.01 s are created from each dataset at each position. The histograms of the time differences in the training and test data are denoted as *P* and *Q*, respectively. Then, the KL divergence between *P* and *Q* is calculated as follows:

(2) $$D_{KL}\left(P||Q\right)=\sum_{i=1}^{25}P\left(i\right)\log\frac{P(i)}{Q(i)},$$

where P(i) and Q(i) are the frequencies of the *i*-th bins of histograms *P* and *Q*, respectively.

When the frequency of a certain bin is empty, the KL divergence cannot be calculated because P(i) or Q(i) becomes 0, which causes an undefined log0 or divide by zero error. To avoid this problem with a general solution, a very small value of 0.0000001 is added to each bin. Finally, the KL divergence between the test and training datasets has been calculated for each body part, the part with the smallest KL divergence is estimated as the load position by the proposed method as follows, where Y denotes the set of positions with attached wearable devices.

(3) $$\underset{y\in Y}{\arg\;\min}D_{KL}(P_{y}\left||Q\right)$$

## 4. Evaluation

This section describes an experiment that we conducted to evaluate the accuracy of estimating device load positions with the proposed method.

### 4.1. Performance Evaluation of R-Peak Detection Algorithm

#### 4.1.1. Experimental Environment

First, we evaluated the performance of the R-peak detection algorithm. The dataset for the evaluation was the MIT-BIH Arrhythmia Database [39], which is available from PhysioNet [40]. It contains 48 sets of ECG data with arrhythmias, which were collected from 47 subjects (25 men aged 32–89 years, and 22 women aged 23–89 years). The length of each set of data is 30 min. The data were recorded at 360 Hz. In this experiment, the sensitivity (SEN) and the positive predictive value (PPV) were used as the evaluation criteria; they are calculated by the following equations:

(4) $$SEN=\frac{TP}{TP+FN}\times 100,PPV=\frac{TP}{TP+FP}\times 100,$$

Here, TP, FN, and FP denote the number of true positives (correctly identified R-peaks), false negatives (missing R-peaks), and false positives (wrongly identified R-peaks), respectively.

The peak position detected by our algorithm is allowed to be within 100 ms of the peak position of the correct answer in the MIT-BIH Arrhythmia Database. We chose this value because it was used in related studies [41,42].

#### 4.1.2. R-Peak Detection Results

For the 48 datasets, Table 1 lists the values of TP, FP, FN, SEN, and PPV, along with the average (AVE) difference between the peak time of the correct answer and that of the detected peak, the standard deviation (SD) of this difference. The values of AVE and SD were calculated from the TP samples.

As seen in the table, with a tolerance range of 100 ms for peak detection, some sessions had SEN and PPV values over 90%. Accordingly, we concluded that the detection algorithm could detect peaks correctly. However, the peaks in some sessions were not detected correctly even within the tolerance of 100 ms. For example, in the case of record ID 210, the maximum value for all the datum is 3.32, but the maximum value from the start of measurement to the 10,000th sample is 1.08. The proposed algorithm uses the maximum and average values of the data during the measurement time to calculate the threshold for peak detection, which is not a suitable approach for record ID 210. In other words, if noise causes the maximum value to be significantly different, the threshold value may not be calculated correctly. In addition, the data for record ID 114 data shows an inverted signal, and we can see in Table 1 that the peaks were not detected correctly. Indeed, the R-peak detection algorithm was implemented under the assumption that the signal was not inverted. On the other hand, the error in the time of the detected peak was less than 3 ms for many sessions. Because the MIT-BIH dataset was recorded at 360 Hz, the time gap between samples is about 2.78 ms. Thus, the detected peaks were off by at most one sample. Therefore, when detection is possible, the R-peak detection algorithm in the proposed method is highly accurate.

Next, Table 2 summarizes the SEN and PPV values for the proposed detection algorithm and other detection algorithms. The results are compared for three records: record ID 100, which was correctly detected; and the two problematic records mentioned above (IDs 114 and 210), which were not correctly detected with the a tolerance of 100 ms.

As seen in Table 2, the detection accuracy for record IDs 114 and 210, in which the R-peaks were not detected correctly, was much lower with the proposed algorithm than with the other algorithms. On the other hand, the results for record ID 100 were similar to those of the other algorithms. Because the proposed method is designed for data collection in advance, problematic data such as record IDs 114 and 210 would not be measured in practice, and peaks would be detected with high accuracy as in record ID 100. However, to deal with a wider range of situations, the algorithms used in previous studies should also be adopted. Accordingly, we will consider improvement of the proposed algorithm and adoption of algorithms from previous studies.

### 4.2. Data Collection

**Figure 5:**
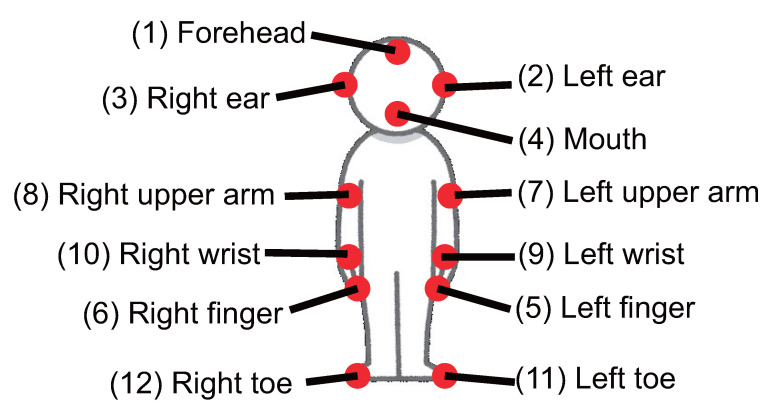
Target body positions.

Five subjects (denoted A, B, C, D, and E; four men (A–D), and one woman (E); average age: 23.0 years) participated in our data collection experiment. Pulse wave sensors were attached to 12 body parts, and an ECG sensor with three electrodes was attached near the heart. As shown by the red circles in Figure 5, the 12 target body parts were the (1) forehead, (2) left ear, (3) right ear, (4) mouth, (5) left hand finger, (6) right finger, (7) left upper arm, (8) right upper arm, (9) left wrist, (10) right wrist, (11) left toe, and (12) right toe.

The ECG and pulse wave data were collected for 3 min at a time, which constituted a session. We collected data for two days with a maximum of two sessions per day. Thus, a total of 3 min × 2 sessions × 2 days × 12 parts × 5 subjects = 720 min of data were collected. Note that the participants were permitted to determine the order in which data were collected from each body part, so the order differed among the participants and sessions. Because the pulse wave sensor was removed between each sessions, its position varied slightly from session to session even on the same body part. The data were collected while the subjects were sitting and wearing clothes.

In this experiment, the pulse was collected using a PulseSensor.com device (https://pulsesensor.com/ accessed on 29 January 2022). The specific pulse sensor used here was a photoelectric pulse wave sensor (PPG sensor). The photoelectric pulse wave method uses an LED to irradiate green light at a wavelength around 550 nm to the skin surface, and the irradiated light reflected by body tissue is received by a photodiode on the skin surface. The ECG was collected using a SHIELD-EKG-EMG device (https://www.olimex.com/Products/Duino/Shields/SHIELD-EKG-EMG/open-source-hardware accessed on 29 January 2022) manufactured by OLIMEX, which is an Arduino compatible shield. The pulse sensor and ECG sensor were connected to an Arduino Uno Rev3, and the pulse wave and ECG were collected using a laptop connected to the Arduino board. The sampling rate was 695 Hz. Note that while the ECG and pulse wave sensors were manufactured by different companies, their measurement times could be synchronized because they were both connected to the Arduino board.

After the data collection, the peak detection, peak time difference calculation, and position estimation were performed offline by a program implemented in Python.

For each session, peak detection was performed, and the time difference between the ECG and pulse wave peaks (hereafter referred to as the peak time difference) was calculated. In the rest of the paper, we refer to Session 1 as S1, Session 2 as S2, Session 3 as S3, and Session 4 as S4. Table A1 lists the age, sex, and average heart rate (bpm) obtained at each body part for each subject during data collection.

### 4.3. Preliminary Analysis of Peak Time Differences

**Figure 6:**
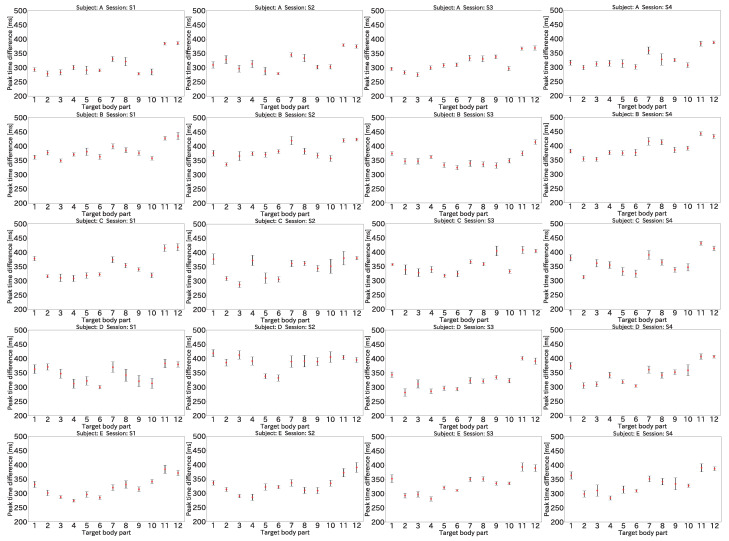
Average and standard deviation of peak time differences.

The average and standard deviation of the peak time difference for each body part and each session for the five subjects are shown in Figure 6. The numbers on the horizontal axis correspond to the numbers of the body part positions shown in Figure 5. A • represents an average time difference, with the error bars indicating the standard deviation.

As seen in Figure 6, the time difference for each body part depended on the distance from the heart. For example, in the case of subject A, the peak time difference for (4) the mouth was approximately 300 ms, whereas the differences for (11) the left toe and (12) the right toe were approximately 380 ms. The peak time differences for the other subjects showed the same trend. This was a natural result of the different distances between the heart and each body part: the mouth is closer to the heart than the a toe is, and the peak time difference of the mouth was thus smaller than that of the toe. In addition, several body parts had similar peak time differences within the same session because their distances from the heart were almost the same. For example, in S3 for subject A, the peak time differences of (5) the left finger and (6) the right finger were 308 and 309 ms, respectively. Similarly, in S2 for subject C, the peak time differences of (11) the left toe and (12) right toe were both 380 ms.

Furthermore, the peak time difference was different in each session even for the same subject. As seen in Figure 6, the peak time differences between S1 and S2 and between S3 and S4 collected on the same day were also different for subjects B, C, and D. For example, for (10) the right wrist of subject D, the peak time difference of S1 was 313 ms, but that of S2 was 405 ms. In another example, for (7) the left upper arm of subject B, the peak time difference of S3 was 339 ms, but that of S4 was 415 ms. These results were due to the fact that the heart rate and blood pressure change moment by moment.

### 4.4. Datasets and Environment

**Figure 7:**
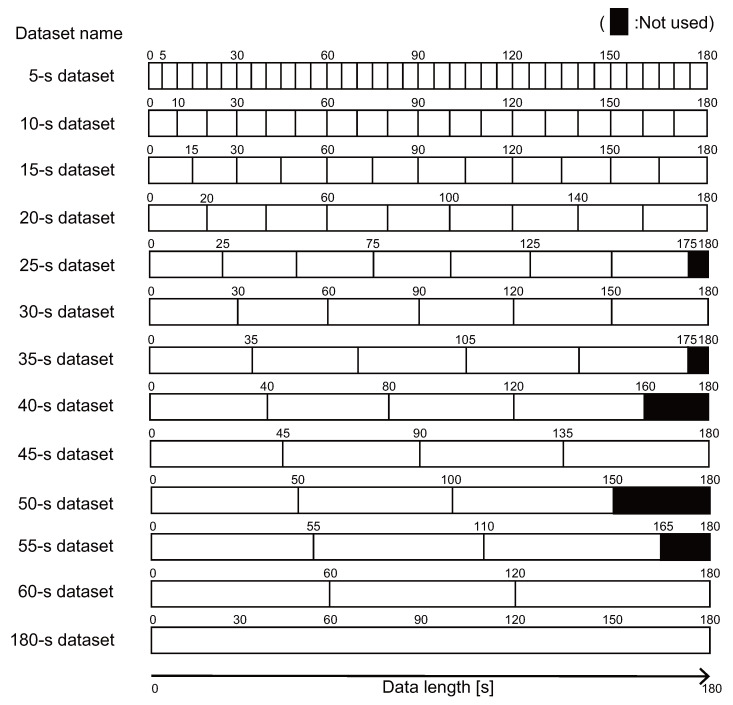
Split datasets used in the evaluation experiment.

We expected that the proposed method’s performance would be affected by the length of the data for training and testing. To understand this effect, we investigated the performance with the data split in the 13 different ways as illustrated in Figure 7. Here, 12 of the splits were created by splitting the 180 s of data into 5 to 60 s intervals in 5 s increments, and the remaining data were the original 180 s data. For example, the 5 s dataset consisted of 36 pieces of 5 s data, and the 40 s dataset consisted of four pieces of 40 s data, with the extra 20 s of data discarded. Table A2 lists the number of peak time differences calculated for each subject, session, and body part.

**Figure 8:**
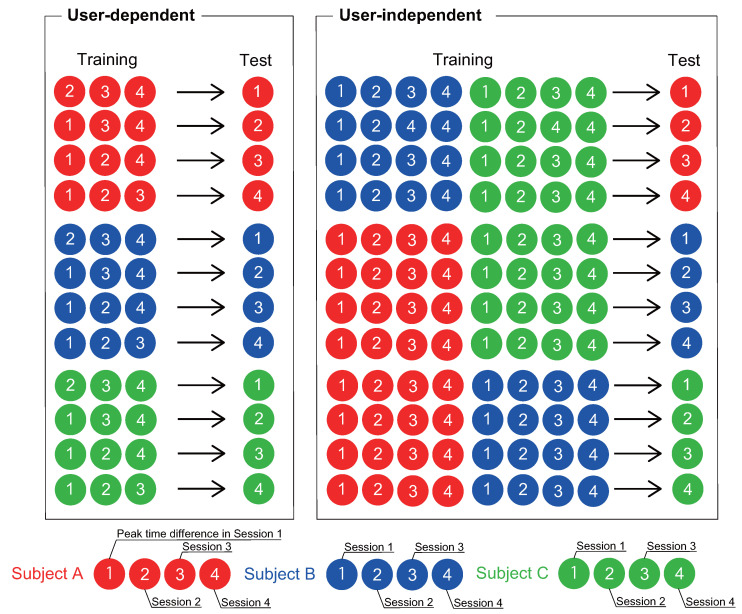
Illustration of two experimental environments using data consisting of four sessions each from three subjects. The circles represent the peak time differences in each session.

Using the data consisting of four sessions each from subjects A, B, and C, we conducted experiments in two different environments, as illustrated in Figure 8 and described below.

- User-dependent: To estimate the sensor position on the same subject but in a different session, we defined a user-dependent environment in which the training and test data came from different sessions for the same subject;
- User-independent: To estimate the sensor positions for different users, we defined in a user-independent environment in which the training and test data were from different subjects.

### 4.5. User-Dependent Experiment

#### 4.5.1. Experimental Environment

The user-dependent experiment was conducted with each subject. The number of target body parts was varied from 2 to 12, and all combinations of each body part were tested, so that ${}_{12}C_{2}=66$ combinations were tested for two body parts. As described above, this experiment was user-dependent in that the training and test data were from the same subjects. The training data were taken from three of the four 180 s sessions, and all peak time differences in the selected sessions were obtained as the training data were learned. The test data were taken from the fourth session and were split 13 different ways, as described above. All possible combinations of training and test data were used.

Because the estimation results were output as many times as the number of pieces of training data, we used the following two methods to narrow down multiple estimation results to one result and obtain the final estimated position.

- Minimum-value method (Mini-method): The body part that showed the smallest KL divergence value in the training data with respect to the input data session was estimated as the load position;
- Majority-vote method (Vote-method): The load position was estimated for each piece of training data, and the final position was estimated by a majority vote. When multiple body parts tied for the highest number of votes, the part with the smallest KL divergence was estimated as the load position.

**Figure 9:**
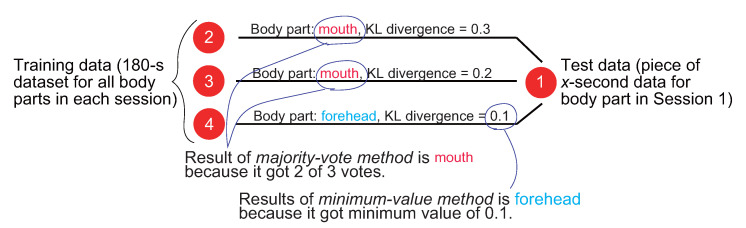
Examples of final load position estimation by the Mini-method and Vote-method.

Hereafter, we referred to these methods as the Mini-method and the Vote-method. Figure 9 illustrates the process of outputting the final results for each method.

In this paper, the evaluation index is the F-value, which is the harmonic mean of the precision and the recall. The closer the F-value is to 1.0, the better the system performance is. The F-value is calculated by Equation (5):

(5) $$\text{F-value}=\frac{2\times \mathrm{precision}\times \mathrm{recall}}{\mathrm{precision}+\mathrm{recall}}$$

By varying the number of target body parts from 2 to 12, we investigated the change in the F-value according to the number of parts. The average F-value was calculated for each combination with the same number of target body parts, and the maximum among these average F-values was used as the final result. For example, given training data from S1 and test data from S1, we wanted to estimate the attachment positions for ${}_{12}C_{2}=66$ combinations of two body parts. First, we calculated the average F-value for each of the 66 combinations. Then, we obtained the maximum value among the 66 calculated average F-values as the final result.

#### 4.5.2. Results for Position Estimation Accuracy When Varying Input Data Length

**Figure 10:**
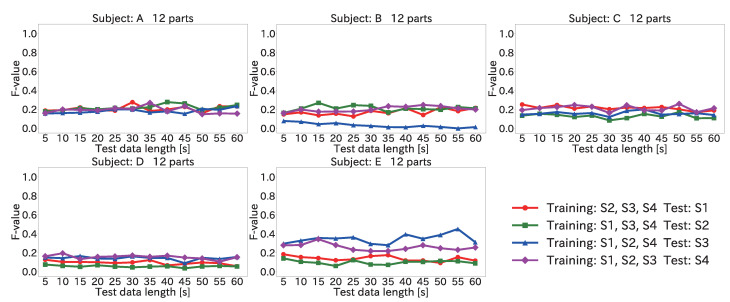
Average F-value when varying the input data length for 12 body parts by using the Mini-method in the user-dependent environment.

**Figure 11:**
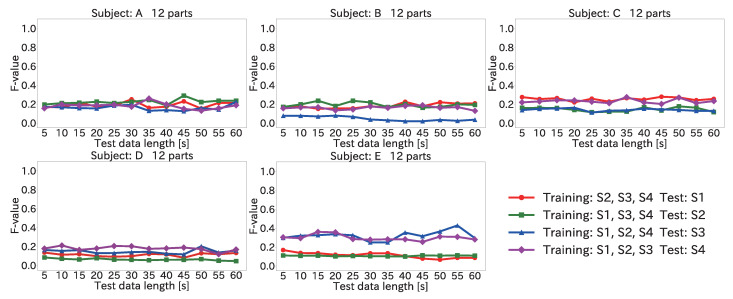
Average F-value when varying the input data length for 12 body parts by using the Vote-method in the user-dependent environment.

Figure 10 and Figure 11 show the average F-value over 12 body parts for each subject and each session when the input data length was varied, by using the Mini-method and the Vote-method, respectively.

As seen in the figures, the average F-value was low regardless of the input data length. This was because the distribution of the peak time differences was different between the training and test data sessions. Specifically, the peak time differences depended on factors such as the subject’s physical condition and blood pressure. For example, for the right wrist of subject D, the modes of the histogram of peak time differences in S1, S2, S3, and S4 were 305, 405, 325, and 355 ms, respectively. When S1, S3, and S4 for subject D were used for training and S2 was used for testing, the distributions of the peak time differences were very different, and the load position could not be estimated correctly. Accordingly, it is necessary to collect data under multiple conditions, such as at rest and after exercise.

The figures also show that the best method depended on the input data length. For example, when the input data length for subject A was 45 s, the Mini-method had a higher F-value in all test data sessions. When the input data length for subject A was 10 s, however, the F-value was higher with the Vote-method when the test data were from S3.

Furthermore, the best method depended on the subject. When the input data length was 60 s, the F-value was higher for subject A with the Mini-method for all test data sessions. In comparison, the F-value for subject D was higher with the Vote-method for test data from S1, S2, and S3 and with the Mini-method for S4. These results indicate that it is necessary to select the best method to obtain a high F-value. An example of how to select the best method is to collect data from one body part before starting load position estimation and then to use the method that can estimate the correct position of that part.

#### 4.5.3. Results for Position Estimation Accuracy When Varying Number of Target Body Parts

**Figure 12:**
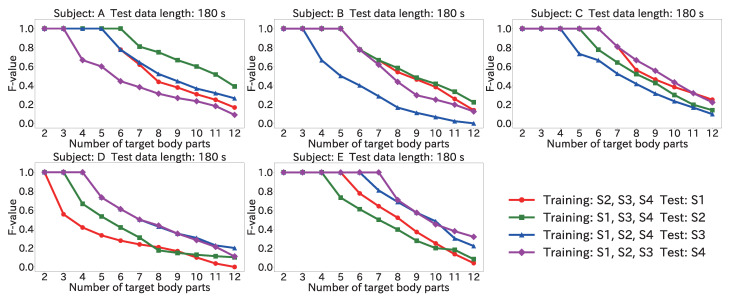
Maximum of the average F-value when varying the number of target body parts with 180 s input data by using the Mini-method in the user-dependent environment.

**Figure 13:**
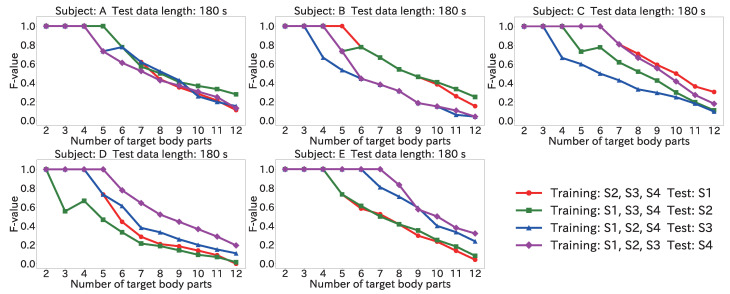
Maximum of the average F-value when varying the number of target body parts with 180 s input data by using the Vote-method in the user-dependent environment.

Figure 12 and Figure 13 show the maximum of the average F-value for each body part when the number of target body parts was varied from 2 to 12 by using the Mini-method and the Vote-method, respectively. The input data length was 180 s for both methods.

For test data from S2 for subject A, the maximum of the average F-value was 1.0 from two to six body parts with the Mini-method, and 1.0 from two to five body parts with the Vote-method. The same result can be seen for test data from S4 for subject D and test data from S1 for subject E. A high F-value could thus be obtained by limiting the number of target body parts. The number of target body parts was lower for the Vote-method here because there were no suitable sessions in the training data. When there was no training data that were suitable for the test data, the peak time difference distributions of the collected data and the wrong target body part were similar. As a result, because of incorrect estimation results, the wrong body part received votes. Accordingly, the Mini-method is more suitable for use in a user-dependent environment.

### 4.6. User-Independent Experiment

#### 4.6.1. Experimental Environment

The user-independent experiment was also conducted with each subject. The training data consisted of 16,180 s sessions for the four subjects, and all peak time differences in the selected sessions were obtained as the training data were learned. The test data were taken from one of the four sessions that was not selected for the training data, and were split into 13 different datasets ranging in length from 5 to 180 s datasets. All possible combinations of training and test data were used. Because there were multiple pieces of training data, the Mini-method and Vote-method proposed in Section 4.5 were used to obtain the final estimated load position. In addition, the number of target body parts was varied from 2 to 12, and all combinations of each body part were tested.

#### 4.6.2. Results for Position Estimation Accuracy When Varying Input Data Length

**Figure 14:**
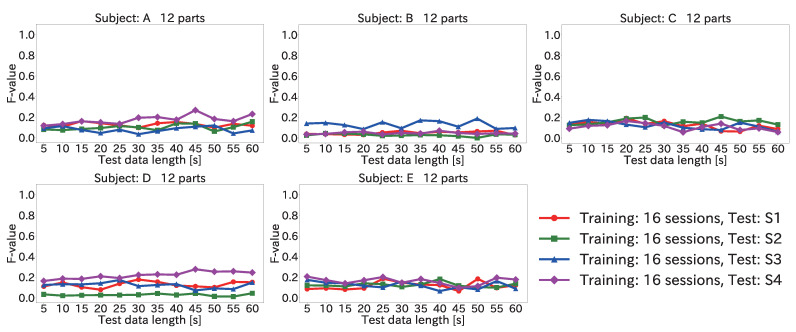
Average F-value when varying the input data length for 12 body parts by using the Mini-method in the user-independent environment.

**Figure 15:**
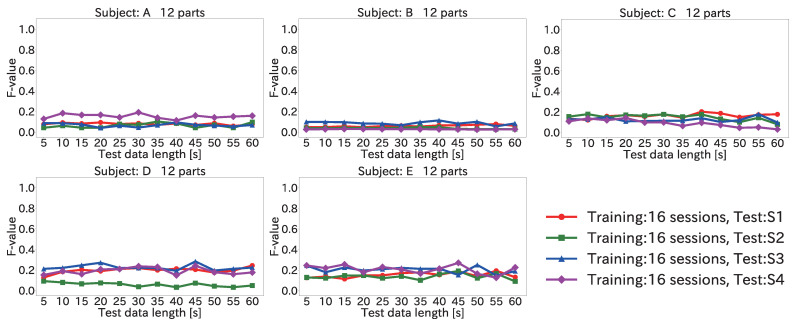
Average F-value when varying the input data length for 12 body parts by using the Vote-method in the user-independent environment.

Figure 14 and Figure 15 show the average F-value over 12 body parts for each subject and each session when the input data length was varied, by using the Mini-method and the Vote-method, respectively.

As seen in the figures, the F-value did not improve significantly even when the input data length was increased. This was because the peak time difference for each body part was different for each subject. For example, the average value of the peak time difference for (12) the right toe in S1 for subject A was 385 ms. In comparison, the average values of the peak time difference for (12) the right toe in each session for subject B were 435, 423, 413, and 432 ms. Because the peak time differences between the training and test data were different, a low F-value was obtained. Accordingly, it is necessary to eliminate the peak time differences between the subjects for each body part.

#### 4.6.3. Results for Position Estimation Accuracy When Varying Number of Target Body Parts

**Figure 16:**
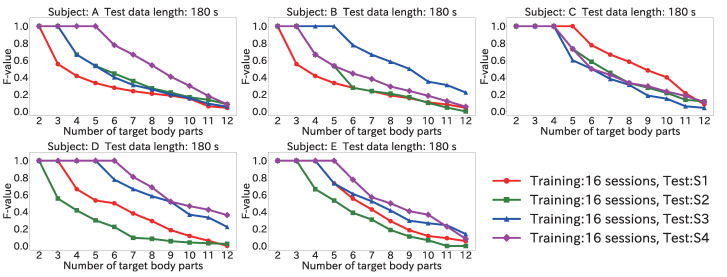
Maximum of the average F-value when varying the number of target body parts with 180 s input data by using the Mini-method in the user-independent environment.

**Figure 17:**
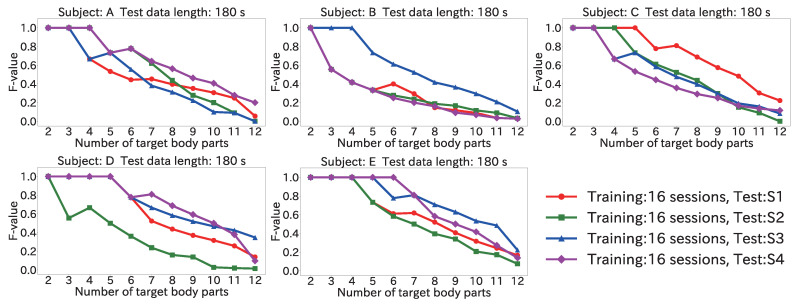
Maximum of the average F-value when varying the number of target body parts with 180 s input data by using the Vote-method in the user-independent environment.

Figure 16 and Figure 17 show the maximum of the average F-value for each body part when the number of target body parts was varied from 2 to 12, by using the Mini-method and the Vote-method, respectively. The input data length was 180 s for both methods.

As seen in Figure 16, for the test data from all subjects, an F-value of 1.0 was obtained from two to five body parts. Furthermore, when the test data were from S4 for subject D, the F-value was 1.0 from two to six body parts. Accordingly, the proposed method can be used even in a user-independent environment by limiting the number of target body parts to about five.

Regarding the method used to obtain the final estimation result, Figure 16 shows that the F-value was 1.0 from two to five body parts by using the Mini-method when the test data were from S4 for subject A. On the other hand, as seen in Figure 17, when the Vote-method was used with test data from S2 and for S4 for subject A, the F-value was 1.0 from two to four body parts. For Subjects B, C, and D, as well, the Mini-method had more target body parts for which the F-value was 1.0. This was because many wrong body parts were estimated, and the number of votes was larger than the number of correct body parts. From the results of Section 4.3, the peak time difference for each body part was different for each subject. For example, the difference for (11) the left toe in S3 for subject A was 366 ms; however, (11) the left toe in S4 for the subject B was 442 ms, and the closest difference to 366 ms was that for (5) the left finger (374 ms). For this reason, misrecognition occurred, and votes were given to the wrong body part. Accordingly, the Mini-method is better for use in a user-independent environment.

### 4.7. Summary of Evaluation

In this evaluation, the F-value was 1.0 for two to five body parts in both the user-dependent and user-independent environments. In addition, the proposed method obtained a high F-value by using the Mini-method to narrow down the number of estimation results to one. The proposed method can thus estimate the load position with high accuracy if the number of target body parts to be estimated is limited and the final result is output by using the Mini-method, regardless of whether data are learned for the user in question.

Here, we compare the accuracy of our method with the accuracies of previous studies on estimating the load positions of wearable devices. Vahdatpour et al. [24] obtained an average accuracy of 84% for 10 positions, including the upper arm; Timo et al. [25] obtained an average accuracy of 89% for seven positions, including the left wrist; and Takata et al. [13] obtained an average accuracy of 90% for 10 positions, including the left wrist. In comparison, the accuracy of the proposed method was as high as an F-value of 1.0 for five parts, which is very high. However, the number of body parts that can be estimated with high accuracy by the proposed method is comparatively small. The great advantage of the proposed method is that it does not require specific actions, such as walking, that the previous studies required. Accordingly, this novel method can provide a certain level of evaluation, but it will need to be improved to achieve a high level of accuracy for many body parts.

## 5. Limitations

This section discusses the limitations of the proposed method.

### 5.1. Estimation of Load Positions with Similar Peak Time Differences

From the results described in Section 4, the peak time differences were similar for body parts that were a similar distance from the heart. For example, the average values of the peak time difference for the left and right toes were both about 400 ms, or nearly the same value for the same body part on the left and right sides. In the evaluation experiment, the subjects were asked to sit. Accordingly, by using a measurement posture in which the peak time difference is different for each body part, the accuracy of the estimated load position will be improved even if the number of target body parts is high. Examples of such measurement postures include raising one arm, lying down, or crossing the arms.

Moreover, the body parts that are prone to be misrecognized depend on the subject. The accuracy may thus be improved by using features other than the peak time difference. For example, the thickness of skin varies from person to person, which causes variations in the measured pulse wave. Therefore, taking the pulse wave amplitude and shape into consideration may improve the accuracy.

### 5.2. Number of Target Body Parts for Load Position Estimation

From the results described in Section 4, the more body parts to be recognized, the lower the accuracy that is obtained for the target body parts. The optimal number of target body parts and the optimal combination of body parts depend on the user and the experimental environment. To increase the number of target body parts, it will be necessary to solve the problem of certain parts having the same peak time differences. In contrast, to limit the number of target body parts, a method of searching for the optimal number and combination of body parts will be needed. One approach would be to collect data from specific body parts in advance and reduce the number of parts that have similar peak time differences.

### 5.3. Dataset Environment

All the data for the evaluation experiment were collected with the subjects sitting, and both the training and test data were collected in the same environment. Accordingly, it will be necessary to evaluate whether the load position can be estimated by collecting the training data at rest and the test data in a state other than sitting. An example of such a state would be a situation in which the heart rate increases, such as after exercise.

In addition, the experimental subjects were young people between the ages of 22 and 25; there were no older subjects. It will thus be necessary to evaluate the performance of the proposed method with data collected from subjects of various ages.

### 5.4. User Dependence

From the results described in Section 4.6, when the number of target body parts was small, a high F-value was obtained by using other subjects’ peak time difference distributions. However, if no other subject is a suitable match, misrecognition may occur. In addition, because the estimation results are output for each training data session, the amount of calculation becomes large when there is a large amount of training data. Accordingly, it will be necessary to create and use a basic common model. There is a technique for calculating the pulse wave velocity (PWV) from the difference in pulse wave arrival times between known body parts, and this technique can be applied to measure health indices, such as the blood vessel age. In particular, the brachial-ankle PWV (baPWV) is a convenient, non-invasive method for measuring pulse waves in the upper arm and ankle [45]. By measuring the PWV between known body parts before using the proposed method, a basic model constructed in advance can be fitted to users according to their ages.

### 5.5. Time Synchronization

In the evaluation experiment, the pulse and ECG sensors were analog-connected to a single Arduino board; therefore, time synchronization was not required. In a practical application, however, the pulse and ECG sensors would have their own processors, and they would transmit sensor data to a computer to calculate the time differences independently via USB or Bluetooth. In that case, the sensor devices would require synchronization. Once synchronization is ensured, the transmission delay and data processing time do not affect the proposed method, because the devices send sensor data with timestamps when analog values are read. Nevertheless, accurate synchronization of multiple devices remains an ongoing issue. In addition, an oscillator would produce a time gap due to frequency stability. A general oscillator with a ±50 ppm frequency stability (SG-8018CB, Seiko EPSON Corp., https://www5.epsondevice.com/en/products/crystal_oscillator/sg8018cb.html accessed on 29 January 2022) would generate a ±180-ms time gap per hour, which would affect the proposed method. Analog–digital conversion also suffers from jitter, i.e., deviation from the true periodicity of a presumably periodic signal; however, the amount of jitter is much less than a single clock cycle and would not affect the proposed system. Further investigations will be required to explore these issues.

## 6. Conclusions

In this paper, we have proposed a method for estimating the load position of a wearable device by using the time differences between ECG and pulse wave peaks.

The proposed method estimates the load position in real time. First, it measures the ECG and pulse wave for a certain time and detects their peaks. Then, it calculates the time differences between those peaks and outputs a load position estimation result by calculating the distance between the distributions of the peak time differences in the training data and the test data.

We conducted an evaluation experiment with four male subjects and one female subject by collecting ECG and pulse wave data at 12 body parts. Because the proposed method outputs as many results as the number of pieces of training data, we applied two methods to narrow down multiple estimation results to one result and obtain the final estimated position. By limiting the number of estimated body parts to about five in both user-dependent and user-independent environments, the proposed method could achieve a high F-value.

In the future, we will define postures that can generate differences in the peak time difference for each body part, and we will verify whether the accuracy of load position estimation is improved by using these postures. We will also examine differences in the age and condition of each user, as well as differences in the user’s situation like after exercise when the heart rate increases. In addition, we will investigate the optimal combination of body parts and develop a method for determining that combination. Finally, we plan to examine how to select another user with suitable training data when there are no data for the user in question, and how to estimate the load position without creating a distribution of peak time differences when there are no suitable training data.

## Figures and Tables

**Table 1:** Results of the R-peak detection algorithm on the MIT-BIH dataset.

Record ID	TP	FP	FN	SEN[%]	PPV[%]	AVE[ms]	SD[ms]
100	2272	3	1	99.96	99.87	1.49	1.54
101	1791	69	74	96.03	96.29	−1.82	1.35
102	2084	3	103	95.29	99.86	−3.92	10.82
103	1999	0	85	95.92	100	1.91	1.29
104	1187	355	1042	53.25	77.98	−4.02	11.82
105	204	262	2368	7.93	43.78	0.23	8.37
106	1272	53	755	62.75	96.0	0.48	3.10
107	1951	150	186	91.30	92.86	−9.60	3.58
108	20	428	1743	1.13	4.46	−7.92	18.91
109	1460	40	1072	57.66	97.33	−3.03	4.54
111	66	106	2058	3.11	38.37	−2.99	5.06
112	968	108	1571	38.13	89.96	0.71	1.27
113	1795	42	0	100	97.71	1.57	1.38
114	69	228	1810	3.67	23.23	10.31	14.16
115	1939	0	14	99.28	100	3.31	1.09
116	1396	159	1016	57.88	89.77	2.33	3.33
117	564	186	971	36.74	75.2	−17.16	6.80
118	610	76	1668	26.78	88.92	3.67	4.55
119	1983	750	4	99.80	77.56	1.86	2.13
121	163	276	1700	8.75	37.13	0.82	2.09
122	2473	2	3	99.88	99.12	0.84	1.68
123	1514	0	4	99.74	100	3.16	1.03
124	1499	93	120	92.59	94.16	−1.68	2.21
200	307	93	2294	11.80	76.75	2.93	2.33
201	364	108	1599	18.54	77.12	2.92	1.11
202	950	0	1186	44.48	100	2.02	1.27
203	183	371	2797	6.14	33.03	−1.52	8.40
205	2561	18	95	96.42	99.30	1.17	2.05
207	18	319	1842	0.97	5.34	0.31	21.63
208	505	148	2450	17.09	77.34	5.66	7.86
209	2804	6	201	93.31	99.79	2.49	1.13
210	2	75	2648	0.08	2.60	5.56	2.78
212	1880	0	868	68.41	100	2.08	1.24
213	3223	97	28	99.14	97.08	0.07	6.36
214	2127	91	135	94.03	95.90	2.49	3.14
215	159	27	3204	4.73	85.48	2.18	2.58
217	341	145	1867	15.44	70.16	−4.52	12.07
219	2119	124	35	98.38	94.47	0.74	2.44
220	2048	0	0	100	100	3.41	1.40
221	2310	83	117	95.18	96.53	1.57	2.25
222	945	4	1538	38.06	99.58	0.88	1.59
223	1984	0	621	76.16	100	4.31	2.04
228	285	156	1768	13.88	64.63	0.54	2.12
230	1856	1	400	82.27	99.95	3.07	1.06
231	1565	2	6	99.62	99.87	−0.42	1.12
232	1183	4	597	66.46	99.66	3.11	1.18
233	2244	45	835	72.88	98.03	0.52	5.25
234	2347	69	406	85.25	97.14	0.70	1.21

**Table 2:** Performance comparison of R-peak detection algorithms.

Record ID	Reference	SEN[%]	PPV[%]
	Proposed method	99.96	99.87
100	Lu et al. [43]	100	99.96
	Gupta et al. [44]	100	100
	Proposed method	3.67	23.23
114	Lu et al.	99.79	94.08
	Gupta et al.	99.95	99.95
	Proposed method	0.08	2.60
210	Lu et al.	98.38	99.92
	Gupta et al.	99.98	99.96

## Data Availability

The data are not publicly available due to using biometric information such as ECG signal and pulse wave.

## Appendix A. Heart Rates for Each Subject

**Table A1:** Age, sex, and average heart rate (bpm) for each subject during data collection.

Body Part	Subject A				Subject B				Subject C				Subject D				Subject E			
	Age 24, M				Age 24, M				Age 22, M				Age 25, M				Age 22, F			
	S1	S2	S3	S4	S1	S2	S3	S4	S1	S2	S3	S4	S1	S2	S3	S4	S1	S2	S3	S4
(1)	71	72	86	78	75	61	69	67	78	60	75	66	77	67	87	77	64	76	68	65
(2)	76	72	79	73	72	66	74	69	70	60	73	65	78	67	85	74	66	73	66	66
(3)	71	73	82	76	74	69	74	63	71	65	70	63	80	63	82	78	62	65	68	65
(4)	72	69	78	81	69	66	71	69	66	62	73	64	79	64	85	73	64	75	67	66
(5)	74	79	72	79	74	69	95	74	69	62	84	66	83	76	95	77	73	83	73	69
(6)	75	80	72	79	72	67	94	69	71	70	73	65	77	77	94	81	74	75	71	64
(7)	74	68	79	72	70	71	99	76	65	69	79	64	83	73	93	77	73	78	68	66
(8)	74	74	76	82	69	70	93	75	70	68	80	63	83	73	92	81	71	74	87	84
(9)	74	66	69	74	61	69	95	72	66	61	77	68	79	74	87	84	70	79	70	70
(10)	75	72	76	77	66	68	93	70	65	62	79	65	83	73	90	78	75	76	71	67
(11)	71	68	80	84	74	77	98	75	65	67	73	65	82	79	83	80	83	79	71	67
(12)	76	71	87	71	70	78	80	69	58	63	74	67	78	79	83	80	81	87	74	67

## Appendix B. Peak Time Differences for Each Subject

**Table A2:** Number of peak time differences obtained for 180 s data.

Body Part	Subject A				Subject B				Subject C				Subject D				Subject E			
	S1	S2	S3	S4	S1	S2	S3	S4	S1	S2	S3	S4	S1	S2	S3	S4	S1	S2	S3	S4
(1)	214	216	260	236	226	185	208	203	236	183	228	200	232	201	262	231	194	229	204	196
(2)	228	218	239	219	217	201	223	209	211	181	220	197	236	201	256	224	201	221	201	200
(3)	213	220	247	230	223	209	223	189	213	195	211	189	240	190	247	236	186	196	204	197
(4)	217	209	234	243	208	199	215	208	200	188	219	194	238	192	255	221	193	227	202	198
(5)	224	237	218	238	224	207	287	223	209	188	254	199	250	228	285	232	221	249	221	207
(6)	226	242	216	239	216	203	282	210	216	211	220	195	233	232	284	243	225	225	213	192
(7)	222	204	237	218	211	215	297	230	197	208	239	192	251	221	281	233	219	234	206	198
(8)	222	222	230	246	209	211	280	224	212	205	241	191	249	219	277	243	215	232	209	203
(9)	223	198	208	224	185	207	285	218	198	185	232	203	237	223	263	252	211	237	212	210
(10)	227	216	228	233	199	205	281	210	194	188	238	196	249	220	271	236	227	230	215	202
(11)	213	205	241	253	224	233	295	224	196	202	221	195	247	237	251	242	248	237	215	201
(12)	228	214	263	215	213	235	241	210	175	189	222	203	235	237	250	240	245	264	222	203
